# Streamlined mail-based methods for large randomised trials: lessons learnt from the ASCEND study

**DOI:** 10.1007/s00125-019-05049-8

**Published:** 2019-11-30

**Authors:** Marion M. Mafham, Louise J. Bowman, Richard J. Haynes, Jane M. Armitage

**Affiliations:** 1 Clinical Trial Service Unit and Epidemiological Studies Unit, Nuffield Department of Population Health, University of Oxford, Richard Doll Building, Old Road Campus, Roosevelt Drive, Oxford, OX3 7LF UK; 2 MRC Population Health Research Unit, Nuffield Department of Population Health, University of Oxford, Oxford, UK

**Keywords:** Aspirin, Cardiovascular, Fatty acid, Methodology, Randomised, Review, Streamlined

## Abstract

Reliable assessment of the effects of an intervention usually requires large randomised trials but such studies are becoming increasingly complex and costly to run. ‘Streamlined’ trials are needed in which every aspect of the trial design and conduct is simplified, retaining only those elements needed to answer the research question and ensure the safety of the individual participants. In this review we discuss how the trial ‘A Study of Cardiovascular Events iN Diabetes’ (ASCEND) was streamlined. The study included a two-by-two factorial design: it assessed the effects of low-dose aspirin and, separately, supplementation with *n*-3 fatty acids on serious vascular events in 15,480 people with diabetes but no overt cardiovascular disease. Other key streamlined design features, such as mail-based recruitment and follow-up, mainly by post, with no in-person visits and use of a run-in period, are also described. We go on to discuss the success of the study and other studies that have employed a similar mail-based approach, and the type of clinical trials that are suitable for mail-based design. Finally, we consider the limitations of the study, and how these could be circumvented in future studies. ASCEND randomised large numbers of eligible participants, achieved good adherence rates and almost complete follow-up at a fraction of the cost of traditional clinic-based trials. Such studies are necessary if researchers are to address the important clinical questions most relevant to improving health.

## Introduction

Reliable assessment of treatment effects requires randomised trials with unbiased assessment of large numbers of outcomes, complete follow-up of all randomised participants and adequate adherence to the allocated treatment. However, randomised studies often include complex procedures that can hinder achievement of these aims. Large trials assessing serious clinical outcomes (Phase 3 or 4 studies) have become so costly that many important research questions go unanswered, while some potentially useful therapeutic agents are not brought to clinical testing [1, 2]. Some investigators have suggested that observational analyses of large, complex, routinely collected datasets can be used to assess the effects of treatments without randomisation. However, such methods can lead to erroneous estimates of treatment effects since differences exist between those treated and not treated that are not captured within the dataset, and the bias resulting from these confounding factors can be as large, if not larger, than the treatment effect itself [3].

To obtain randomised trial evidence at feasible costs, ‘streamlined’ methods are needed. In such trials, every aspect of the study design and procedures is simplified as much as possible, retaining only the elements needed to answer the central research question and keep the individual participants safe [4]. Strategies to improve efficiency in trials include conduct in a small number of countries, simple eligibility criteria, limited data collection and the adoption of risk-based monitoring [5, 6]. However, despite these measures, the cost of running large streamlined clinic-based outcome studies [7] remains high and such studies can often only be carried out in partnership with the pharmaceutical industry. ‘Registry trials’ in which participants are identified, randomised and followed-up within an existing registry can be highly cost-effective [8, 9] and have been used in a number of settings, including percutaneous coronary procedures [10–12] and acute myocardial infarction [13]. However, such designs are not suitable or possible for many research questions, and other approaches to streamlining trials are needed.

‘A Study of Cardiovascular Events iN Diabetes’ (ASCEND) was designed to answer two important questions [14, 15]: for people with diabetes but no overt cardiovascular disease, is the risk of serious vascular events safely reduced by (1) low-dose aspirin or (2) supplementation with *n*-3 fatty acids? ASCEND used streamlined methods to achieve randomised trial evidence at as low a cost as possible. This review describes eight design features of the trial (Table 1) and considers how the lessons learnt can inform future studies.

**Table 1:** Key streamlined design features of the ASCEND trial

Feature	Traditional design	ASCEND design
Factorial design	Under-used	Two-by-two factorial design enabled two questions to be answered
Recruitment and assessment	Clinic-based with multiple sites	No clinics. Mail-based recruitment and follow-up, mainly by post
Study organisation	Multiple countries	Conducted within the UK
Run-in period	Under-used in many trials	Two month placebo run-in managed by postal mail improved later adherence
Eligibility criteria	Complex criteria requiring blood or other tests and review of medical notes	Simple criteria that can be determined from participant self-report
Baseline assessments	Complex assessments, including collection of biological samples in all participants	Limited data collection Optional blood and urine sampling
Outcome adjudication	Double adjudication by Clinical Outcomes Committee	Single adjudication using pragmatic procedures
Post-trial follow-up	Seldom undertaken and rarely possible in international trials	Low-cost post-trial follow-up through linkage with routine healthcare records

## Streamlined design features of ASCEND

### Two-by-two factorial design

By using a two-by-two factorial design, ASCEND was able to assess the effects of two independent treatments on serious vascular events within the same population [14, 15]. Provided the effects of each treatment are moderate, such factorial designs have little impact on the statistical power of each analysis [16] and bring obvious efficiency savings by addressing two questions in one trial. It is frequently suggested that they are vulnerable to an interaction between the two treatments under study; however, if real, such interactions would be important to detect, and such a design is the most efficient way to do so. Despite the use of factorial designs in clinical trials for decades, this valuable method of increasing efficiency in randomised studies remains under-used.

### Mail-based trial design with no in-person visits

ASCEND mainly used mail by post for recruitment, assessment of eligibility, consent, supply of study treatment and collection of follow-up information, i.e. there were no participant visits [14, 15]. Other trials of aspirin, vitamins and fish oil supplementation have successfully used similar mail-based methods [17–21]; however, most recruited healthcare professionals (Table 2).

**Table 2:** Large randomised trials conducted by mail

Study (year completed)	BDS 1988 [18]	PHS 1989 [19]	WHS 2005 [17]	WACS 2007 [20]	ASCEND 2017 [14,15]	VITAL 2018 [21]
Intervention	Aspirin	Aspirin, β carotene	Aspirin, vitamin E	Vitamin C, vitamin E, β carotene	Aspirin, *n*-3 FA	Vitamin D *n*-3 FA
Population	UK doctors	US doctors	Female health professionals	Women with, or at high risk of CVD	Diabetes	General population
Invitations	~20K	0.26M	1.7M	53K^a^	0.4M	~9M
Randomised	5139	22,071	39,876	8171	15,480	25,871
Mean or median follow-up (years)	~5	4.8	10.1	9.4	7.4	5.3

^a^Invited female health professionals who had already expressed interest in research

BDS, British Doctors Study; CVD, cardiovascular disease; FA, fatty acid; PHS, Physicians Health Study; VITAL, VITamin D and OmegA-3 TriaL; WACS, Women’s Antioxidant Cardiovascular Study; WHS, Women’s Health Study

Mail-based trial designs result in a substantial cost saving over clinic-based methods but are not suitable for interventions where in-person procedures are necessary to assess eligibility, deliver the intervention, assess physical or biochemical outcomes or monitor individual participant safety (Table 3). However, careful planning can avoid some visits. For example, in ASCEND, baseline blood and urine samples were collected from two-thirds of the randomised cohort by mailing a sample collection kit and consent form and asking the participant to attend their local general practice for the blood to be taken. Samples were then mailed to the central laboratory [22, 23]. This also allowed baseline blood pressure, height and weight to be measured by the practice nurse. With advances in technology, an increasing number of assessments could be made using participants’ smart phones or wearable devices, such as the assessment of cardiac rhythm [24]. Alternatively, if in-person baseline assessments are required, follow-up by postal mail or e-mail can still achieve substantial efficiency savings.

**Table 3:** Characteristics of clinical trials suitable for mail-based design

	Mail-based design suitable	Mail-based design unsuitable
Healthcare system	National healthcare system with access to centralised medical records available	Local healthcare systems comprising multiple providers with no centralised medical records
Eligibility	Eligibility can be identified from routine healthcare records and/or patient report (e.g. prior stroke or myocardial infarction)	Eligibility requires detailed review of medical notes (e.g. heart failure with reduced ejection fraction) or recent blood results (e.g. kidney function)
Intervention	Suitable for administration in remote settings (e.g. oral medication)	Requires administration in a healthcare setting (e.g. devices) or in-person participant education (e.g. injectable therapies)
Safety monitoring	No requirement for regular blood or other tests to monitor safety	Requires regular blood or other tests to monitor safety
Outcomes	Can be identified from routine healthcare records or self-report (e.g. cardiovascular events)	Require in-person assessment (e.g. measurement of physical function)

### Study location

ASCEND was conducted in a single country, which reduced cost and complexity. International collaboration is often necessary when recruiting individuals with a rare disease, but many trials in common diseases involve multiple countries. This is often driven by requirements of regulatory authorities to obtain trial data within particular countries, marketing strategies for new drugs and a perception that results from international trials are more generalisable than those from trials conducted in a single country. However, the proportional effects of treatment are not likely to vary by country unless there are substantial differences in disease pathophysiology or healthcare provision that modify the effects of the treatment. Obtaining the necessary regulatory and ethics committee approvals for international studies adds substantial complexity and delay [25], and operational procedures must be arranged for each country.

Furthermore, by recruiting participants entirely within the UK, low-cost long-term follow-up can be conducted via linkage to electronic health records (EHRs). This is particularly relevant for the aspirin vs placebo comparison, given the recently raised hypothesis that aspirin may reduce long-term cancer risk [26]. In ASCEND, pre-specified cancer analyses will be undertaken in 2022 and 2027 based on cancers identified from the UK Cancer Registry and from coded hospitalisation data.

### Eligibility criteria

Complex eligibility criteria, frequently requiring manual review of the participant’s medical record and trial-specific blood or other tests, such as imaging, can have unhelpful consequences. The procedures required to implement such protocols can be time consuming and expensive and participants be may inappropriately excluded, limiting generalisability. To be eligible for ASCEND, potential participants had to fulfil just six criteria. They had to be at least 40 years of age at the time the screening questionnaire was sent, have a clinical diagnosis of diabetes mellitus, no clear indication for aspirin (e.g. diagnosed occlusive arterial disease) and no clear contraindication to aspirin (e.g. recent gastrointestinal haemorrhage or peptic ulcer; use of anti-coagulant; or history of aspirin allergy). Participants had to be free of severe comorbid disease that might limit their ability to remain in a long-term study, and both the participant and their doctor had to be uncertain about whether antiplatelet or *n*-3 fatty acid therapy conferred worthwhile benefit.

### Run-in period

Maintenance of adherence to the study intervention in randomised trials is critical to ensure adequate statistical power to detect plausible moderate effects. Although ethical standards require trial participants to be free to stop any aspect of participation at any time, every effort should be made to randomise only those participants who are likely to remain adherent for the duration of the study. A run-in period prior to randomisation during which treatment (active or placebo, depending on the circumstance) is taken provides a useful method of identifying participants prior to randomisation who are likely to be non-adherent so that they can be excluded. A run-in period may be particularly valuable in mail-based studies since research staff are unable to make a detailed face-to-face assessment of likely long-term adherence. In ASCEND, more than 40% of eligible individuals entering the placebo run-in period were not randomised, predominantly because they were unwilling to continue in the trial or failed to respond to questionnaires. If these individuals had been randomised immediately after assessment of eligibility, the resulting reduction in treatment adherence would have led to catastrophic loss of statistical power [23].

### Limited baseline assessments

In many trials, complex baseline assessments can include long questionnaires, clinical measurements, collection of biological samples and other tests. The rationale for these assessments includes confirmation of eligibility, characterisation of the cohort and to allow subgroup analyses. While some trials may require biochemical or physical measures to determine eligibility, or as part of the outcome assessments, a random sample of the population is often sufficient. In ASCEND, baseline data were limited to self-reported information about diabetes history, cardiovascular risk factors and non-study medication use. The non-mandatory blood and urine samples collected at the participants’ local general practices, which were mailed to the central laboratory, enabled further characterisation of around two-thirds of the randomised cohort, which allowed assessment of the effects of the treatment in various subgroups defined by laboratory results (e.g. HbA_1c_). If this approach is used in a large randomised trial, the proportion of individuals without a valid sample at baseline should be equally distributed between the treatment groups, so that subgroup analyses can simply include a separate subgroup for those individuals without a valid result.

### Outcomes adjudication

In a typical clinic-based trial, adjudication of cardiovascular outcomes often does not alter the main findings but does consume substantial resource [27]. In ASCEND, the ability of participants to accurately report cardiovascular and bleeding events, the key primary, secondary and safety outcomes, was unknown and so such outcomes were adjudicated. Provided adjudication is undertaken blind to treatment allocation, misclassification of small numbers of events does not introduce bias and has little effect on statistical power [3]. Therefore, a streamlined approach was taken, with limited collection of supporting documents from the participants’ general practitioners (GPs) and adjudication of each event by a single trained adjudicator blind to treatment allocation. Unrefuted participant-reported events (i.e. those for which the adjudication procedure was unable to confirm or refute the event because of lack of information) were included in the analysis. The validity of this approach for future studies depends on the outcomes considered. For some outcomes, such as hypoglycaemia, confirmation from GP records may not be useful and for outcomes that are not well reported by participants, inclusion of unrefuted cases might not be appropriate. Analyses assessing the impact of adjudication on the main ASCEND trial results will be detailed in a later publication.

## Lessons learnt from the practical implementation of the ASCEND study design

The lessons learnt from implementing the streamlined design features of ASCEND are summarised in Table 4.

**Table 4:** Lessons learnt from the practical implementation of the ASCEND study design

Study aspect	ASCEND process	Lessons learnt	Future possibilities
Identification of potentially eligible individuals	Searches of healthcare data (e.g. retinopathy registries) and GP lists	Obtaining permissions and managing formats for multiple datasets caused delay	Search of National datasets (e.g. Hospital Episode Statistics in England and Wales) Linkage with additional datasets to refine eligibility prior to invitation
Invitation to take part	Large numbers of centrally mailed invitation letters sent by post	Response rate low	Requirement to identify large numbers of potentially eligible individuals
Assessment of eligibility	Participant self-report on mailed screening questionnaire	Substantial resource needed to process and check completed questionnaires	Online or app-based questionnaires
Consent	Mailed consent form with telephone support from study nurses and doctors	Changing regulatory framework may require a ‘prior interview’	Online or app-based consent materials with interactive questions supported by Skype or telephone interview
Biological samples	Optional blood collection (at local general practice, mailed to a central laboratory)	Postal delays meant delayed processing of samples Response rate ~70%	Linkage to routine healthcare lab results
Collection of follow-up data	Mailed or online 6-monthly follow-up questionnaires Systematic chasing of non-responders	Substantial resource needed to process and check questionnaires and clarify inconsistencies/missing answers	Online or app-based participant questionnaires Ascertainment of outcomes from routine healthcare data
Maintaining adherence	Coordinating centre staff contacted those wishing to stop treatment to encourage continuing	Required substantial resource at the coordinating centre	Direct discussion with participants likely to still be required

### Participant identification and invitation

The ASCEND recruitment procedures are described in detail elsewhere [22]. Potentially eligible patients with diabetes were identified from electronic searches of centrally held diabetes registers (e.g. for retinopathy screening) in collaboration with local investigators, for which permission was obtained under UK national privacy legislation (Section 251 of the Health and Social Care Act). This allowed details of identified patients to be transferred securely to the coordinating centre in order to generate invitations on behalf of the collaborating investigator. Participants were also identified from GP-held registries, facilitated by local research networks. Practices searched their electronic records for eligible individuals with diabetes and mailed them an invitation pack (including an invitation letter, screening questionnaire including the consent form, information leaflet and freepost envelope) that had been provided preassembled by the coordinating centre. Obtaining a sufficient number of potentially eligible patients for invitation required collation of 58 separate diabetic register datasets and liaison with 785 general practices.

### Assessment of eligibility, consent and pre-randomisation run-in period

Around 400,000 people were mailed an invitation pack by post and around one third returned a valid screening form. Screening forms were logged, checked and scanned using optical character recognition software. Eligibility for the study was determined from the participant’s responses on the screening questionnaire. The questionnaire contained binary questions [e.g. ‘Has a doctor ever told you that you had a stroke or ministroke (sometimes called TIA)?’] along with some questions requesting a written description from the participant. These written responses were reviewed by a clinician, with further details sought by telephone as needed. The screening and other ASCEND questionnaires are available online [23].

Consent required the person to confirm and sign that they had understood the implications of entering the trial having read the information leaflet and having had questions relating to trial entry, if any, addressed by telephone. Future trials will need to establish cost-effective methods of establishing valid consent without an in-person visit and regulations made sufficiently flexible to allow this. Such methods could include an online or app-based video describing the study, with a participant-completed quiz to test understanding and support available by telephone, e-mail or skype to answer questions [28].

Consenting and eligible individuals entered a 2 month run-in period and were sent a run-in pack of medication containing placebo aspirin and placebo *n*-3 fatty acids along with a medication information sheet and a copy of their signed consent form. An automated letter informed their GP of the patient’s potential enrolment and asked them to contact the coordinating centre if they did not wish their patient to be randomised. Approximately 2 months after entering the run-in period, participants were sent a randomisation questionnaire to confirm eligibility and willingness to continue.

The overall ‘conversion rate’ (i.e. the proportion of those invited who were randomised) was low. Around one-third of those invited returned a valid screening form but, of these, only about one fifth entered the run-in period (6% of those invited) since the majority of respondents declined to take part. Approximately 40% of those entering the run-in period were not randomised [22, 23]. Overall, 4% of those invited (15,480 participants) were randomised. Future studies using similar invitation methods need to ensure that large numbers of potentially eligible people are available to invite.

Few trials that use traditional clinic-based recruitment collect data on how many potential participants were approached for enrolment. The conversion rate from invitation to randomisation in ASCEND was similar to the other mail-based studies included in Table 2 and to a recently completed clinic-based cardiovascular trial which recruited 8000 participants in the UK by mailed invitation [29]. In ASCEND, available data for those who declined to take part was limited but those living in more deprived areas and older individuals were less likely to return a screening questionnaire indicating willingness to take part [22]. Future studies recruiting from within a single national dataset could undertake more detailed comparisons between those who did and did not take part. However, while differences in social deprivation, age, comorbidity or other factors between trial participants and the target patient population will affect the likely absolute harms or benefits, the proportional effects of treatment are likely to be generalisable.

Managing the mail-based recruitment process required considerable resources at the coordinating centre, including a 24 h freephone service for participants or their doctors. Bespoke computer programs were developed to enable and control all aspects of the trial. Dedicated trial managers, nurses and clinicians performed manual checks of the data and contacted the participants for clarification where necessary.

Recruitment to ASCEND took 6 years but could be faster in future. Identification of potentially eligible individuals from a single national dataset would bring substantial efficiency savings. Linkage with additional datasets, such as prescribing data, could refine eligibility prior to invitation and use of online or app-based forms to collect information about eligibility would eliminate many of the manual checks that were required to process the ASCEND screening forms.

### Biological samples

Approximately 2–4 weeks after entering the run-in period, participants were sent a blood and urine sample kit and asked to take it to their GP or other usual phlebotomy service. The kit included an information leaflet, a blood collection form (which included a section for recording of blood pressure, pulse, height and weight by the practice nurse) and a consent section (which included a request for consent for long-term storage of samples for future research). Samples were returned to the coordinating centre by mail. Previous stability studies have shown that a wide range of analytes (including HbA_1c_, lipids and cystatin C) and genetic polymorphisms can be reliably measured in mailed samples at ambient temperature [30]. Sixty-three per cent of randomised participants returned a usable blood and/or urine sample within 4 days of sample collection and provided consent for analyses. Those who returned a sample were generally representative of the whole study population [23].

In future, linkage to routine laboratory data could increase efficiency and save money. In an ongoing registry trial in haemodialysis patients, local hospital blood results are obtained via the UK Renal Registry [31], while in a diabetes trial in Scotland (Clinical Trials.gov Identifier NCT03439345), linkage to the Scottish Care Information Diabetes Collaboration allows the study to collect routine blood and urine results.

### Collection of follow-up information

Every 6 months, participants were sent a short questionnaire asking about clinical events and admissions to hospital, adherence to the study treatments and use of anticoagulants or non-study antiplatelet agents. Each returned questionnaire was checked and scanned, with automated checks for missing or inconsistent responses. Any written descriptions of events or reasons for stopping the study treatment were reviewed by a study clinician and coded using bespoke software with clarification sought from the participant as necessary. Once coding was complete, details of any serious adverse events reported by participants were populated into the relevant tables within the database. Unless the participant or their doctor had requested that they stop their study treatment, further packs were mailed every 6 months. Serious adverse event reports could also be created by the coordinating centre clinicians if contacted directly by the participant or their doctor.

Throughout the study, the response to the 6-monthly participant follow-up questionnaire was generally over 90%. However, achieving these response rates required considerable work. Participants were sent up to two reminder questionnaires, following which, if no response was received, staff at the coordinating centre made several attempts to contact the participant by phone before mailing a standard letter. If necessary, a call was made to an ‘alternative contact’ whose details were requested on the randomisation questionnaire. If participants still failed to respond, follow-up information was requested from their GP. In addition, the work involved in checking and processing paper follow-up forms was substantial. During the trial an online follow-up questionnaire was developed, which included in-built validation to reduce errors and inconsistencies. Participants who provided a valid e-mail address were given the option of receiving an e-mail link to a secure online version of the follow-up questionnaire. By the end of the study about a quarter of those who were still completing follow-up questionnaires were doing so online.

Future studies should aim to use participant-completed electronic data capture with either online or app-based questionnaires with or without additional use of linked EHR data for outcome ascertainment. In the ongoing Aspirin Dosing: A Patient-centric Trial Assessing Benefits and Long-term Effectiveness (ADAPTABLE) study, a streamlined web-based trial comparing two doses of aspirin in people with prior cardiovascular disease, vascular and bleeding events are assessed from participant reports supplemented by routine hospital claims data, with further investigation reserved for those events where a discrepancy exists [32]. However, future trials in countries with a single health provider or with good record linkage systems could potentially rely entirely on linkage to the EHRs, as is planned for an ongoing trial of allopurinol vs placebo in 5000 people with ischaemic heart disease in the UK [33]. Systems developed by regulatory agencies to link large numbers of health datasets for post-marketing surveillance, such as the EU-ADR web platform [34] and the FDA Sentinel Initiative [35], could be used to obtain outcomes for trials and embedded randomised trials are already part of the FDA Sentinel Initiative [35].

However, several hurdles remain before EHRs can replace clinical-based or questionnaire-based data collection for trials. First, careful validation must be undertaken for each outcome. Most cardiovascular outcomes assessed in ASCEND are accurately captured in the EHRs [36], but some outcomes (e.g. hypoglycaemia or transient ischaemic attack) may be difficult to assess from routinely collected data. For trials of investigational medicinal products, it may be necessary to receive data in a timely fashion to meet regulatory reporting requirements. Furthermore, open access to trial data may be limited by the data sharing agreements with the providers of linked healthcare data. In ASCEND, routinely collected data about cancer, cause of death and admissions to National Health Service (NHS) hospitals in the UK are available for almost all randomised participants and further publications will assess whether comparable results can be obtained using only EHR data.

### Maintaining adherence with study treatment

In ASCEND, any participant who expressed a wish to stop the study treatment or reported low adherence on their questionnaire was contacted by telephone to encourage continuation where appropriate. Participants who stopped the aspirin or placebo treatment were encouraged to continue with the *n*-3 fatty acids or placebo capsule and vice versa. Engagement was maintained with participants using regular newsletters and a study website. Using these procedures, weighted average adherence to study therapy while at risk of an event was 70% for the aspirin or placebo tablet and 76% for the *n*-3 fatty acids or placebo capsule over the mean follow-up duration of 7.4 years [14, 15], which is comparable to many clinic-based trials. Although future studies may make use of app-based or online communications and reminders to encourage adherence, it is likely that personal discussion with individuals to address their concerns will still be required to ensure good rates of adherence.

### Verification of participant identity

A key challenge for mail-based studies is the verification of the participant’s identity and the authenticity of participant-completed responses. In ASCEND, potentially eligible participants were identified within routine healthcare records and therefore identifiers, such as date of birth, were known prior to invitation. For each questionnaire, participants were asked to record their date of birth as a verification check and visual inspection of the signature was undertaken by the coordinating centre staff. Future studies, particularly those in which data are collected online or via mobile device applications, must develop systems to verify the identity of respondents.

## Conclusions

The key streamlined design features of ASCEND allowed a robust result to emerge at relatively low cost (estimated at less than £10 million [23]). However, future studies could recruit large numbers of participants and collect high-quality data more efficiently, for example, by using EHR data for both identification of participants and outcome ascertainment, and by collecting participant-reported data by online or app-based questionnaires. ASCEND has demonstrated that streamlined methods can obtain high-quality randomised trial evidence, sufficient to meet regulatory requirements and directly influence national and international guidelines [37]. Hopefully similar approaches will be adopted by other researchers, since trials need to become less complex and costly to run if important clinical questions most relevant to improving health are to be answered.

## Abbreviations

ASCEND: A Study of Cardiovascular Events iN Diabetes
EHR: Electronic health record
GP: General practitioner
